# Use of Fast Gamma Magnetic Stimulation Over the Left Prefrontal Dorsolateral Cortex for the Treatment of MCI and Mild Alzheimer's Disease: A Double-Blind, Randomized, Sham-Controlled, Pilot Study

**DOI:** 10.3389/fneur.2021.729872

**Published:** 2021-09-09

**Authors:** Alberto José Mimenza-Alvarado, Sara Gloria Aguilar-Navarro, Francisco M. Martinez-Carrillo, Alma E. Ríos-Ponce, Gabriel Villafuerte

**Affiliations:** ^1^Department of Geriatric Medicine & Neurology, Instituto Nacional de Ciencias Médicas y Nutrición Salvador Zubirán, Mexico City, Mexico; ^2^Clínica Coyoacán, Mexico City, Mexico; ^3^Plan de Estudios Combinados en Medicina, Facultad de Medicina, Universidad Nacional Autónoma de México, Mexico City, Mexico

**Keywords:** brain stimulation, low intensity, fast gamma magnetic stimulation, Alzheimer's disease, mild cognitive impairment, gamma oscillation

## Abstract

**Background:** Alzheimer's disease (AD) animal models have shown a reduced gamma power in several brain areas, and induction of these oscillations by non-invasive methods has been shown to modify several pathogenic mechanisms of AD. In humans, the application of low-intensity magnetic fields has shown to be able to produce neural entrainment at the magnetic pulse frequency, making it useful to induce gamma frequencies.

**Objective:** The aim of this study was to assess if the application of fast gamma magnetic stimulation (FGMS) over the left prefrontal dorsolateral cortex would be a safe and well-tolerated intervention that could potentially improve cognitive scores in subjects with mild cognitive impairment and mild AD.

**Methods:** In these randomized, double-blind, sham-controlled study, participants were assigned to either receive daily sessions two times a day of active or sham FGMS for 6 months. Afterward, measurements of adverse effects, cognition, functionality, and depression were taken.

**Results:** Thirty-four patients, 17 in each group, were analyzed for the primary outcome. FGMS was adequately tolerated by most of the subjects. Only four patients from the active FGMS group (23.52%) and one patient from the sham FGMS group (5.88%) presented any kind of adverse effects, showing no significant difference between groups. Nevertheless, FGMS did not significantly change cognitive, functionality, or depressive evaluations.

**Conclusion:** FGMS over the left prefrontal dorsolateral cortex applied twice a day for 6 months resulted to be a viable intervention that can be applied safely directly from home without supervision of a healthcare provider. However, no statistically significant changes in cognitive, functionality, or depression scores compared to sham stimulation were observed.

**Clinical Trial Registration:**www.ClinicalTrials.gov, Identifier: NCT03983655, URL: https://clinicaltrials.gov/ct2/show/NCT03983655.

## Introduction

Gamma oscillations are rhythmic changes of the brain's electrical activity in a broad band of frequencies that range between 25 and 135 Hz; these oscillations can be subdivided into slow gamma (25–60 Hz) and fast gamma (60–135Hz) oscillations (1). Gamma oscillations have been found to be involved in several cognitive processes, such as sensory integration, selective attention, and retrieval of memories (2); because of this, several neurological disorders, including mild cognitive impairment (MCI) (3) and Alzheimer's disease (AD) (4), have shown aberrant behavior in the gamma band of the oscillatory activity of the brain (1, 4).

MCI is a progressive disorder diagnosed when an individual presents loss of cognitive abilities beyond what would be expected given his or her age and educational background, but it does not cause functional impairment (5). AD is a progressive disorder that starts as amnestic MCI that gradually evolves into dementia due to progressive neuronal degeneration and loss, which produces an eventual destruction of cognition, personality, and the ability to function independently even in daily life tasks (6).

AD animal models have shown a reduced gamma power in several brain areas such as hippocampus and prefrontal cortex; restoring normal gamma rhythms by different mechanisms, such as magnetic (7), auditory (8), and visual stimulators (9), has proved to reduce cognitive symptoms, inflammation, and amyloid deposition in these same animal models. However, all the gamma-restoring strategies tested in AD models and human subjects have been performed within the slow gamma range, even if fast gamma range has shown to be related to working memory in humans (10), letting the possible effects of fast gamma-restoring strategies unexplored.

Currently, there are several non-invasive brain stimulation methods that could be used to induce or restore gamma oscillations. Between these methods, transcranial magnetic stimulation (TMS) is a very well-known method that uses electromagnetic induction in order to generate an electric field inside the brain; however, because of the high intensity of the currently used magnetic fields (near 1 T), it is unsafe to apply high-intensity magnetic pulses at fast gamma frequencies (11). Recent research has shown that very small magnetic fields (0.001 T) can also alter the physiology of the brain (12); therefore, we decided to employ the application of low-intensity magnetic fields, which have shown to be able to produce neural entrainment at the magnetic pulse frequency (13).

Here, we hypothesized that the application of small magnetic fields (5 gauss) at fast gamma frequencies over the left prefrontal dorsolateral cortex, an important node for working memory (10, 14) that has been targeted with positive outcomes in previous MCI and AD studies (15, 16), would be a safe and well-tolerated intervention that could potentially improve cognitive scores in subjects with MCI and mild AD.

To prove our hypothesis, we performed a pilot, double-blind, sham-controlled clinical trial, to assess safety and the possible cognitive effects of twice daily application during 6 months of fast gamma magnetic stimulation (FGMS) over the left prefrontal dorsolateral cortex.

## Methods

### Study Design

We performed a parallel randomized sham-controlled proof-of-concept clinical trial with a 1:1 allocation. The study was approved by the research and ethics board of the National Institute for Medical Sciences and Nutrition “Salvador Zubirán” (CONBIOÉTICA-09-CEI-011-20160627) and registered at the clinicaltrials.gov (NCT03983655). The study was originally designed to explore possible cognitive changes in a bigger sample size; however, because of COVID-19, the main outcome of the study was changed, and recruitment was stopped before sample size completion to safeguard the health of participants.

### Participants

All participants were assessed for eligibility for the trial during their consultation in the memory clinic in a tertiary-level University Hospital in Mexico City during the period of March 2019 to March 2020 (see **Figure 2**). For eligibility, all subjects had to be over 65 years old and had to be diagnosed with MCI or mild AD by an attending geriatrician/neurologist and a neuropsychologist; participants were also classified into two groups (MCI and AD) according to their performance in neuropsychological evaluation and current clinical criteria. For the diagnosis of AD, the criteria of the “Diagnostic and Statistical Manual of Mental Disorders Version 5” (17) and the criteria of the National Institute of Neurological and Communicative Disorders and Stroke—Alzheimer's Disease and Related Disorders Association (NINCDS-ADRDA) (17, 18) were used, while MCI diagnosis was established according to Petersen's criteria (19). To determine the severity of the cognitive impairment, the Clinical Dementia Rating (CDR) score was used, a scale that quantifies the severity of symptoms with scores ranging from 0 to 3, helping differentiate MCI (CDR = 0.5) and mild AD (CDR = 1) (20). All subjects also needed to have a stable dementia medication for at least 3 months before starting any intervention and no cognitive training. All included subjects had to have a structural MRI of a maximum of 3 months before their inclusion in the study; medial temporal lobe atrophy was assessed with Scheltens score, a 5-point rating scale with scores ranging from 0 to 4 points, where higher scores indicated more atrophy (21). Subjects with uncontrolled medical conditions, diagnosis of major depressive disorder, metal implants, previous history of seizures, and previous utilization of any brain stimulation devices were excluded from the study. After determining their eligibility, patients were invited to participate in the study and were asked to participate voluntarily and sign an informed consent.

Participants were evaluated twice, at enrollment and after the 6 months of intervention. In each evaluation, subjects were interrogated for any adverse effect and completed a standardized neuropsychological assessment.

### Intervention

Subjects were randomized to receive either daily sessions two times a day of FGMS for 6 months or daily sessions two times a day of sham FGMS for 6 months. The device used in this study was designed and manufactured by Actipulse Neuroscience (Boston, MA, USA); it is portable, can be used at home without medical supervision, and works by passing electric current into a coil to generate a rapidly changing magnetic field at fast gamma frequency. The coil had a circular shape with a 6-cm diameter and 50 turns of copper; it was also mounted over a headband to assure that it was positioned correctly by the patient.

Subjects were instructed by a researcher on how to position the coil over the F3 coordinate of the 10–20 EEG system (left prefrontal dorsolateral cortex), and an illustrated manual with the instructions was also provided to assure that the coil was correctly positioned. Also, the device included a wireless sensor that allowed researchers to evaluate treatment compliance for each patient.

**Figure 1:**
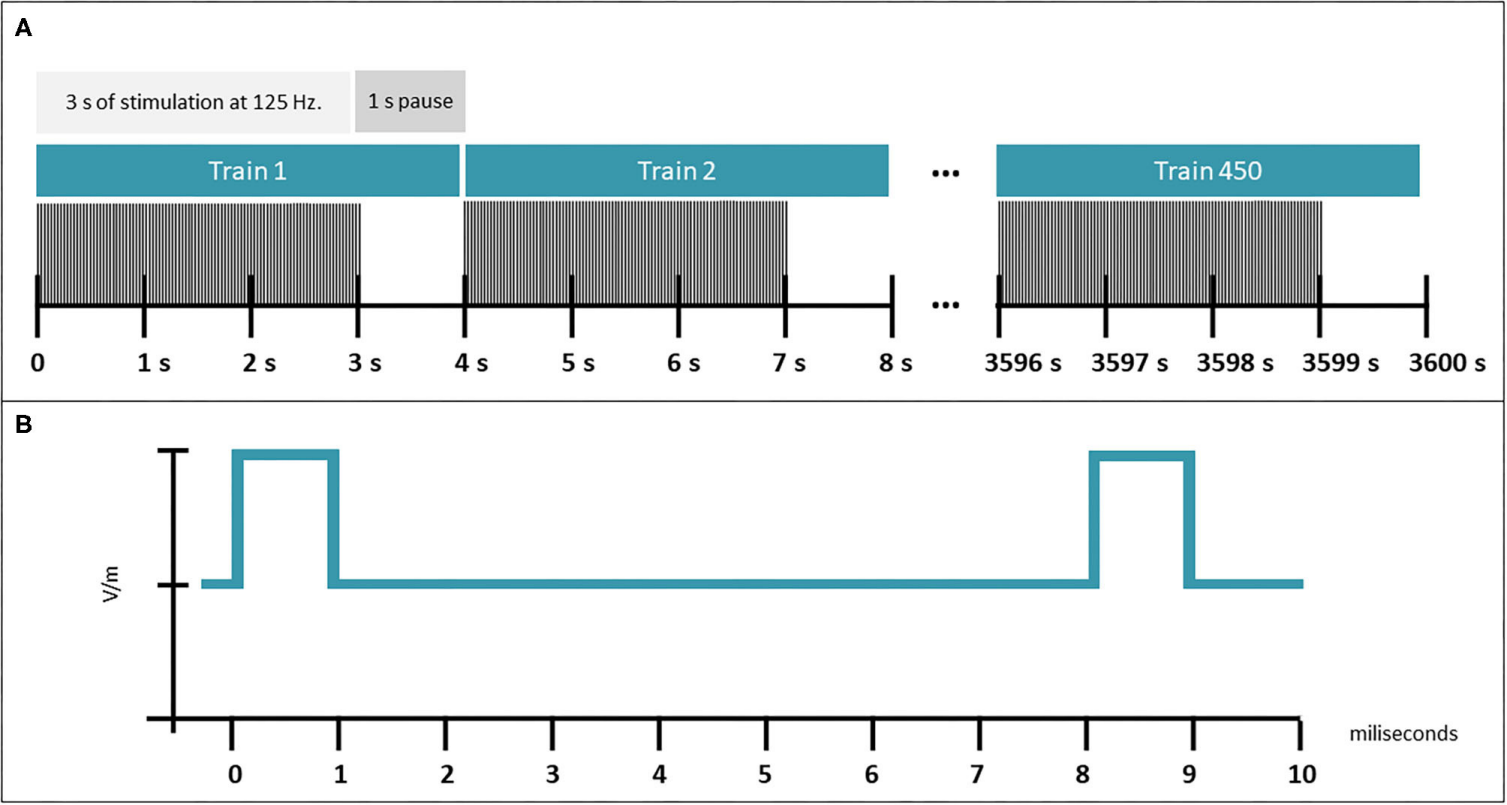
Characteristics of FGMS. **(A)** Pattern of the magnetic field in trains, consisting of 3-s bursts of pulses at fast gamma frequency (125 Hz) alternated with 1-s without stimulation for 450 trains. **(B)** Pulse pattern delivered.

The coil of the device emitted a patterned magnetic field in trains, consisting of 3-s bursts of pulses at fast gamma frequency (125 Hz) alternated with 1 s without stimulation (see Figure 1 for more details about the stimulation pattern); a total of 450 trains (30 min of stimulation) were applied in each session. Each pulse had an approximate magnetic field intensity of 0.5 mT. Adherence was assessed weekly by a blinded researcher through a computerized program, which registered signals emitted by the device when it was connected and turned on; subjects with adherence lower than 80% were eliminated from the study.

### Randomization

A block randomization was used to assign devices to perform any of the two possible interventions. Eight blocks with a fixed size of 10 were created using a computer random number generator. One researcher, who had no interaction with research subjects or other researchers, knew which device corresponded to active or sham intervention and the actual block size. The sham and the active devices were identical for the exception that one device did not emit any kind of magnetic field from the coil. As the intensity of the magnetic field is low, no sensory cues were evoked by the stimulation that could compromise blinding from the research on the subject side.

Each device was labeled from 1 to 80 and were assigned consecutively to each patient entering the trial. Researchers that were involved in patient recruitment, capacitation, and evaluation were blinded to the kind of device they were providing to each subject. Patients and caregivers were also blinded to the kind of device they were using.

### Assessments

The primary outcome was the difference between the proportion of patients suffering any kind of adverse effects between groups during the whole trial. Adverse effects were captured continuously during the study and at the last evaluation performed. For the continuous evaluation, patients and caregivers were instructed to report telephonically to their attending physician any kind of side effects; then, the physician would capture the symptoms and determined if an appointment was necessary to evaluate the symptoms and their cause. Also, in each appointment performed, a systematic interview looking for adverse effects was performed. All adverse effects were reported in the database where clinical outcomes were also captured. For the secondary outcomes, cognition was measured by the Alzheimer's Disease Assessment Scale–Cognitive Subscale (ADAS-Cog), Frontal Assessment Battery (FAB) (22), semantic and phonetic Verbal Fluency Test (sVFT & pVFT, respectively), and Montreal Cognitive Assessment (MoCA) (23); daily living functionality measured by the Katz Index of Independence in Activities of Daily Living (ADL) (24) and the Lawton-Brody Instrumental Activities of Daily Living (iADL) (25); and depression measured by the Geriatric Depression Scale Short Form (GDS-15) (26). All secondary outcomes were measured by the researchers at baseline and 6 months after starting stimulation.

### Statistical Analysis

Baseline characteristics were reported as means and standard deviations. To assess differences between baseline characteristics in the sham and active FGMS groups, chi-square or Mann–Whitney *U*-test was performed, depending on the type of variable. The primary analysis was performed using a per-protocol approach; the proportion of patients suffering from any kind of adverse effect was compared between groups using a Fisher test.

All secondary outcomes were analyzed with a one-way ANCOVA, in which baseline scores were used as a covariate, postintervention scores as the dependent variable, and the intervention (FGMS vs. sham FGSM) as the independent variable. All statistical analyses were performed using IBM SPSS statistical software. Figures were designed with GraphPad prism for windows.

## Results

**Figure 2:**
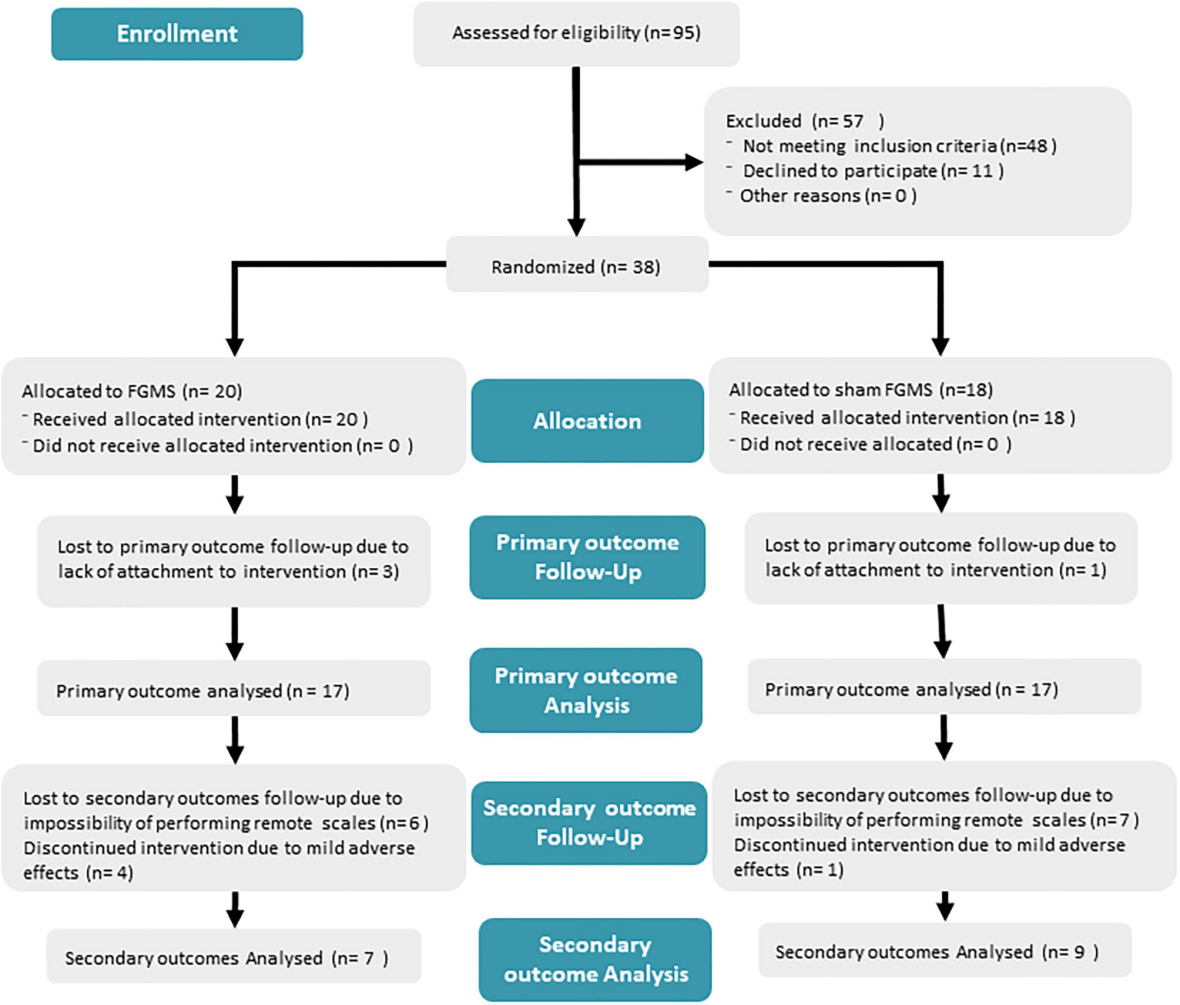
Diagram of the method from recruitment to analysis. The diagram explains the number of subjects in each step and the population per group.

A total of 95 subjects were screened for eligibility; 38 met eligibility criteria and were randomized between May 2019 and March 2020 (see Figure 2). A total of 20 subjects were allocated to the active FGMS group and 18 to the sham FGMS group. Four subjects (three in the active FGMS and one in the sham FGMS) were excluded from the primary and secondary analyses due to lack of at least 80% of attachment to intervention. During the 6 months of the intervention, four subjects presented adverse effects in the FGMS group, while just one subject presented adverse effects in the sham FGMS group. These five subjects were eliminated from the study once the adverse effects were reported and, thus, were not included in the secondary analysis. For the secondary analysis, six subjects from the active FGMS group and seven subjects from the sham FGMS were lost due to impossibility of performing accurate remote evaluation for cognitive scales. In the end, seven subjects from the active group and nine subjects from the sham group were included for secondary outcome analysis.

Baseline demographic and clinical characteristics such as age, sex, baseline scores, and relevant history of comorbidities were measured for the subjects included in primary analysis and are described in Table 1. To evaluate differences in these variables, Mann–Whitney *U*-test and chi-square test were performed depending on the type of variable. Except for the prevalence of stroke, no statistically significant differences were found.

**Table 1:** Baseline characteristics and evaluations of participants in both the sham FGMS and active FGMS groups.

**Characteristic**		**Sham FGMS** **(*n* = 17)**	**Active FGMS** **(*n* = 17)**	***p*** **-value**
Sex – no. (%)	Male	9 (52.94)	14 (82.35)	
	Female	8 (47.06)	3 (17.65)	0.067
Age – years (SD)		82.24 (8.066)	80.47 (8.375)	0.865
Patients with antidementia drugs - no. (%)		5 (29.41)	4 (23.52)	0.697
MTA score. (SD)		1.5 (0.65)	1.375 (0.72)	0.623
Disease stage (%)	MCI	5 (29.41)	5 (29.41)	
	Mild AD	12 (70.59)	12 (70.59)	1
Scores (SD)	ADAS-Cog	13.18 (4.966)	14.10 (5.516)	0.760
	MoCA	17.76 (4.855)	18.29 (4.413)	1
	FAB	12.41 (2.373)	11.24 (3.153)	0.193
	pVFT	8.29 (3.981)	9.43 (5.185)	0.739
	sVFT	11.41 (5.328)	15 (4.772)	0.064
	ADL	5.59 (0.618)	5.82 (0.529)	0.274
	iADL	5.65 (2.317)	6.06 (2.076)	0.610
	GDS	2.53 (1.546)	2.88 (2.998)	0.865
Comorbidities (%)	DM2	2 (11.0 76)	4 (23.53)	0.368
	Hypertension	8 (47.06)	10 (58.82)	0.492
	Hypercholesterolemia	5 (29.41)	10 (58.82)	0.084
	Hypothyroidism	5 (29.41)	4 (23.53)	0.697
	Depression	3 (17.65)	5 (29.41)	0.419
	VI	14 (82.35)	14 (82.35)	1
	AI	10 (58.82)	8 (47.06)	0.492
	CAD	2 (11.0 76)	5 (29.41)	0.203
	Cancer	2 (11.0 76)	4 (23.53)	0.368
	Stroke	5 (29.41)	0 (0)	0.015
Patients that presented any adverse effect		1 (5.88)	4 (23.53)	0.146

*SD, standard deviation; MTA, medial temporal lobe atrophy; DM2, Type 2 diabetes mellitus; VI, visual impairment; AI, auditory impairment; CAD, coronary artery disease*.

### Primary and Secondary Outcomes

Thirty-four patients, 17 in each group, were analyzed for the primary outcome. Only four patients from the active FGMS group (23.52%) and one patient from the sham FGMS group (5.88%) presented any kind of adverse effects. Because of the small sample size, Fisher's exact test was run; there was not a statistically significant difference in proportions of 0.17, *p* = 0.335.

From the active FGMS group, the most common reported adverse event was sensory perception disturbances (three subjects reported visual disturbances and one subject reported experiencing tinnitus) and headache (one subject). From the sham FGMS group, one patient reported visual alterations that were ultimately related to a background condition. All adverse effects in the active FGMS were mild, transitory, and remitted without any medical intervention.

**Figure 3:**
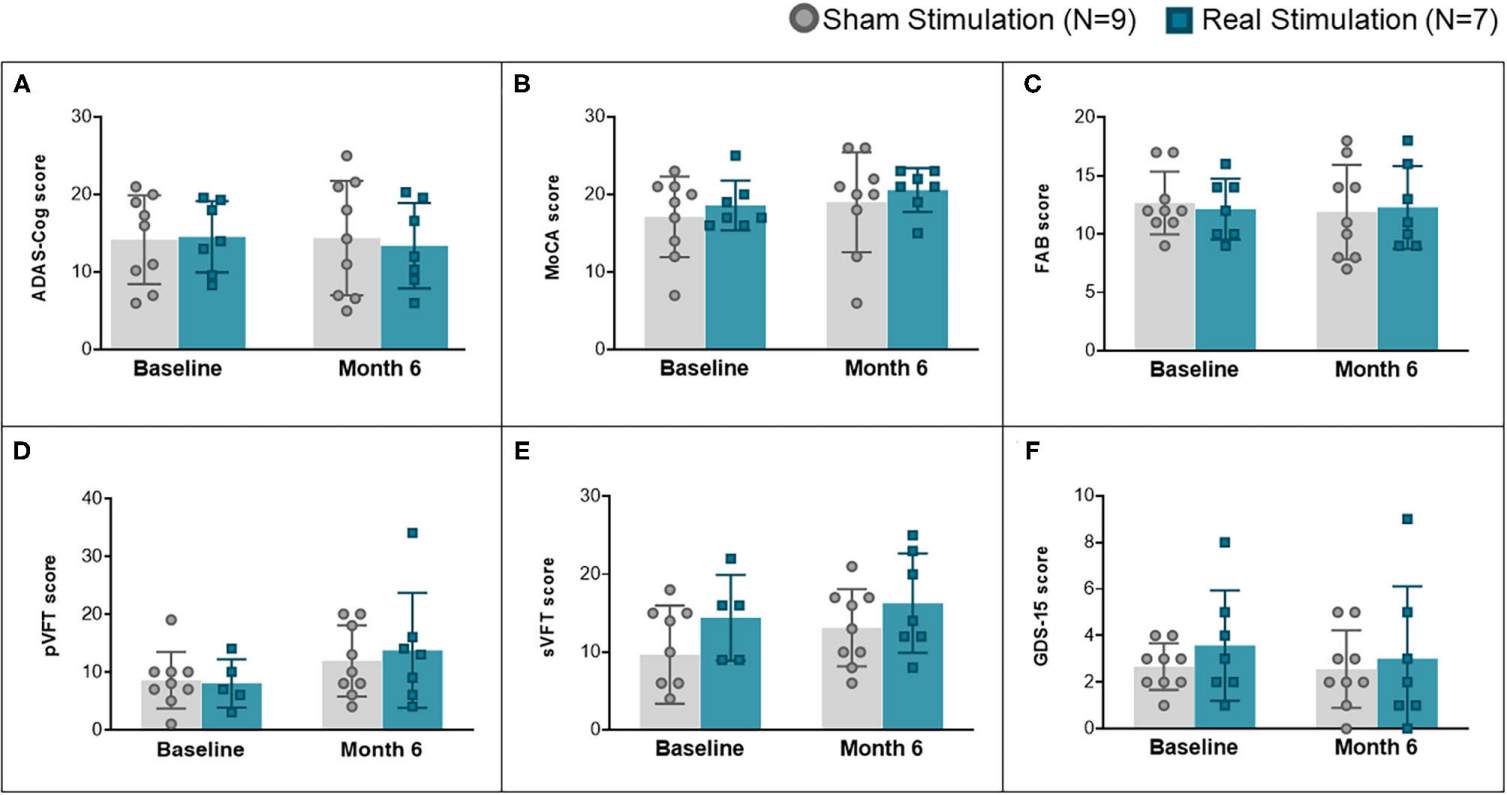
Changes in secondary outcome scores over time, including all cognitive assessments and depression assessment. **(A)** Comparison of ADAS-Cog score at baseline and after 6 months of treatment. **(B)** Comparison of MoCA scores at baseline and after 6 months of treatment. **(C)** Comparison of FAB scores at baseline and after 6 months of treatment. **(D)** Comparison of pVFT scores at baseline and after 6 months of treatment. **(E)** Comparison of sVFT scores at baseline and after 6 months of treatment. **(F)** Comparison of GDS-15 scores at baseline and after 6 months of treatment.

Regarding secondary outcomes, each variable was analyzed using a one-way ANCOVA to determine the effect of FGMS on postintervention scores. After adjusting for preintervention scores, there was no statistical significant differences in ADAS-Cog score, *F*_(1, 13)_ = 0.790, *p* = 0.390, partial η^2^ = 0.057; FAB score, *F*_(1, 13)_ = 0.306, *p* = 0.590, partial η_2_ = 0.023; sVFT score, *F*_(1, 13)_ = 0.250, *p* = 0.627, η^2^ = 0.022; pVFT score, *F*_(1, 13)_ = 0.821, *p* = 0.384, η^2^ = 0.069; MoCA score, *F*_(1, 13)_ = 0.002, *p* = 0.962 η^2^ = 0.000; ADL, *F*_(1, 13)_ = 0.210, *p* = 0.654, η^2^ = 0.016; iADL score, *F*_(1, 13)_ = 3.017, *p* = 0.106, η^2^ = 0.188; and GDS-15 score, *F*_(1, 13)_ = 0.547, *p* = 0.473, η^2^ = 0.040. Because of the lack of statistical significance, *post hoc* analyses were not performed. Results and analysis performed to secondary outcomes are described in Figure 3 and Table 2.

**Table 2:** Secondary outcomes in baseline and after 6 months of treatment of participants in both the sham FGMS and active FGMS groups.

	**Score**		**Baseline**	**6 months**	***p-*** **value**
Secondary outcomes	ADAS-Cog (SD)				0.390
		Sham	14.167 (5.712)	14.389 (7.384)	
		Active	14.543 (4.586)	13.4 (5.507)	
	MoCA (SD)				0.962
		Sham	17.11 (5.183)	19 (6.442)	
		Active	18.57 (3.207)	20.57 (2.820)	
	FAB (SD)				0.590
		Sham	12.67 (2.693)	11.89 (4.045)	
		Active	12.14 (2.610)	12.29 (3.546)	
	pVFT (SD)				0.384
		Sham	8.56 (4.876)	11.89 (6.133)	
		Active	8.00 (4.183)	13.71 (9.945)	
	sVFT (SD)				0.627
		Sham	9.67 (6.305)	13.11 (4.961)	
		Active	14.40 (5.505)	16.29 (6.396)	
	ADL (SD)				0.654
		Sham	5.56 (0.726)	5.56 (0.726)	
		Active	5.86 (0.378)	5.86 (0.378)	
	iADL (SD)				0.106
		Sham	5.67 (2.550)	5.67 (2.693)	
		Active	5.71 (2.215)	6.57 (1.397)	
	GDS (SD)				0.473
		Sham	2.67 (1)	2.56 (1.667)	
		Active	3.57 (2.370)	3 (3.109)	

*SD, standard deviation*.

## Discussion

Here, we present the results of twice daily application of FGMS over the left dorsolateral prefrontal cortex for 6 months in an elderly population with MCI and mild AD.

Regarding safety and usability, FGMS was adequately tolerated by most of the subjects. Only mild adverse effects were reported, and these effects remitted spontaneously without medical intervention. An important finding is that it is feasible to apply FGMS for a prolonged period (twice daily for 6 months) directly from the house of the patient. Non-invasive neuromodulation devices have shown promising results in treating neurodegenerative disorders (27); however, most of the current studies apply the neuromodulation intervention in a clinical setting for a limited period of time (27), which could affect real-world efficacy and compliance for patients and caregivers. As only three subjects in the active FGMS and one in the sham FGMS did not complete the prespecified 80% of sessions, current results show that long-term application of FGMS directly from home is feasible and has a high compliance rate.

In our sample, FGMS did not change the ADAS-Cog score after 6 months of daily application compared to sham stimulation. ADAS-Cog has been used for several years as the gold standard measurement of cognition in dementia populations; however, its utilization in subjects with MCI remains controversial, mainly because its ability to detect relevant changes in the initial stages of dementia has been discussed (28). Because of the small sample size, the possibility that ADAS-Cog was not sensitive enough to detect small changes in the studied population remains.

Other secondary outcomes were not modified by FGMS; cognitive measurements (FAB, MoCA, pVFT, and sVFT) did not achieve statistically significant differences between groups. We choose several different measurements of cognitive functions to cover a broad range of clinical changes; interestingly, the FAB score showed a crossover interaction in which the effect of time on the score was opposite depending on the group the subjects belonged to; unfortunately, because of the small sample size, statistical significance was not achieved; further trials with adequate sample size focusing on frontal clinical changes could be regarded in a future, especially in dementia with frontal involvement such as frontotemporal dementia.

Both groups showed an improvement in some cognitive measurements (MoCA, pVFT, and sVFT). This is a rather uncommon result: the studied populations tend to have cognitive decline with time. Several factors could have provoked the observed improvement in both sham and real FGMS group. Ito et al. (29) used data from several clinical trials to model the placebo response in AD; they found that with short time of interventions (6 months), some improvement falls within the 90% predicted cognitive response, which could explain our results. Longer periods of intervention and follow-up would be required to assess if this response pattern in both groups is maintained or not.

Several parameters of the stimulation could be modified for future trials testing this technology. We decided to use gamma frequencies at the fast range because fast gamma frequencies have shown to be relevant in diverse cognitive functions, especially in working memory (30) and memory encoding (31, 32); however, recent evidence shows that slow gamma frequencies at 40 Hz could be a more adequate stimulation frequency than faster frequencies (33). Entrainment ability of external magnetic pulses has been tested up to slow gamma range; this is mainly due to the difficulty of measuring fast gamma oscillations in a non-invasive way. More studies using FGMS and deep EEG recordings are needed to affirm that FGMS has an entertainment effect in humans.

Another parameter that could explain the lack of clinical changes is the intensity of the applied magnetic field. While the magnetic fields used in the present study are over the threshold for a biological effect (7, 34), the intensity of the magnetic field may not have been enough to assure a perdurable biological effect. Most evidence that affirms that weak magnetic fields are able to cause neural entrainment come from studies using magnetic fields of around 20-fold smaller than current TMS devices (35); in the present study, we used a magnetic field 200 times smaller than current TMS devices. This intensity was used because similar intensities of magnetic fields have shown to modify important pathological mechanisms of neurodegeneration in animal models such as an increase in plasticity (36), an increase in adult neurogenesis (37), and diminishing tau hyperphosphorylation (7). Translating animal findings into human neuromodulation trials encompasses great difficulties, and adjusting the proportional intensity of the magnetic field used in an animal brain to a human brain remains an important methodological challenge. Future trials using gamma magnetic stimulation should take this into consideration.

Lastly, the stimulation site could also be important for the lack of statistical results. Most of repetitive TMS trials in dementia population have used as stimulation site the left prefrontal dorsolateral cortex (27); the rationale of this decision is that the prefrontal cortex has been shown to be selectively affected in AD (38) and is a cortical region that is near and accessible through the skull (39). However, prefrontal cortex pathological changes in early stages of AD are heterogeneous and not as well-described as pathological changes in other areas such as atrophy in medial temporal lobe structures (40). More trials using FGMS focusing on different cerebral regions, such as the temporal lobe, could have different clinical results.

Our results should be regarded with caution as several issues can be noted that could have influenced our results. First, the trial was underpowered due to a small sample size; this was mainly due to the lack of follow-up in our secondary outcomes. Second, we did not include objective biomarkers as inclusion criteria for this trial, and thus, the possibility that our sample was composed of a heterogeneous sample of MCI and other types of mild dementia subjects remains. Third, the follow-up of the current trial was limited only to 6 months; while it is a considerable time frame, neurodegenerative disorders such as MCI and mild AD could demonstrate clinical changes that may have occurred after the last measurement of the variables remains. Also, immediate effects of FGMS were not assessed in this trial; a recent study showed that transcranial alternate current stimulation at slow gamma frequencies was able to improve episodic memory just after finishing stimulation (24). Our trial aimed to encounter long-term changes in scores, and we cannot discard the possibility that FGMS changes, acutely, cognitive traits. Lastly, AD has a higher prevalence in certain specific populations (e.g., women, people with history of depression), and the response to FGMS in these specific populations was not assessed in the current study. Future trials of FGMS should take into consideration the several limitations of our study.

In conclusion, FGMS over the left prefrontal dorsolateral cortex applied twice a day for 6 months resulted to be a viable intervention that can be applied safely directly from home without supervision of a healthcare provider in an elderly population with MCI and mild AD. However, in this small sample, FGMS did not statistically significantly change cognitive, functionality, or depression scores compared to sham stimulation.

## Data Availability Statement

The raw data supporting the conclusions of this article will be made available by the authors, without undue reservation.

## Ethics Statement

The studies involving human participants were reviewed and approved by Research and ethics board of National Institute for Medical Sciences and Nutrition Salvador Zubirán. The patients/participants provided their written informed consent to participate in this study.

## Author Contributions

All authors listed have made a substantial, direct and intellectual contribution to the work, and approved it for publication.

## Funding

Actipulse Neuroscience provided the devices and financed this study.

## Conflict of Interest

The authors declare that GV and AR-P are currently working as part of the Science Department of Actipulse Neuroscience. Actipulse Neuroscience provided the devices and financed this study. Actipulse Neuroscience did not play any role in the study design; collection, analysis, and interpretation of the data; as well as writing of the report and decision to submit the article for publication. The authors declare that the research was conducted in the absence of any commercial or financial relationships that could be construed as a potential conflict of interest.

## Publisher's Note

All claims expressed in this article are solely those of the authors and do not necessarily represent those of their affiliated organizations, or those of the publisher, the editors and the reviewers. Any product that may be evaluated in this article, or claim that may be made by its manufacturer, is not guaranteed or endorsed by the publisher.
