# Supplementary material for: Use of Fast Gamma Magnetic Stimulation Over the Left Prefrontal Dorsolateral Cortex for the Treatment of MCI and Mild Alzheimer's Disease: A Double-Blind, Randomized, Sham-Controlled, Pilot Study
Source: Front Neurol. 2021 Sep 9;12:729872. doi: 10.3389/fneur.2021.729872 (PMC8458744; doi:10.3389/fneur.2021.729872)
Supplement: Supplementary file 1 [file Data_Sheet_1.docx]

Supplementary Material

# Supplementary Figures


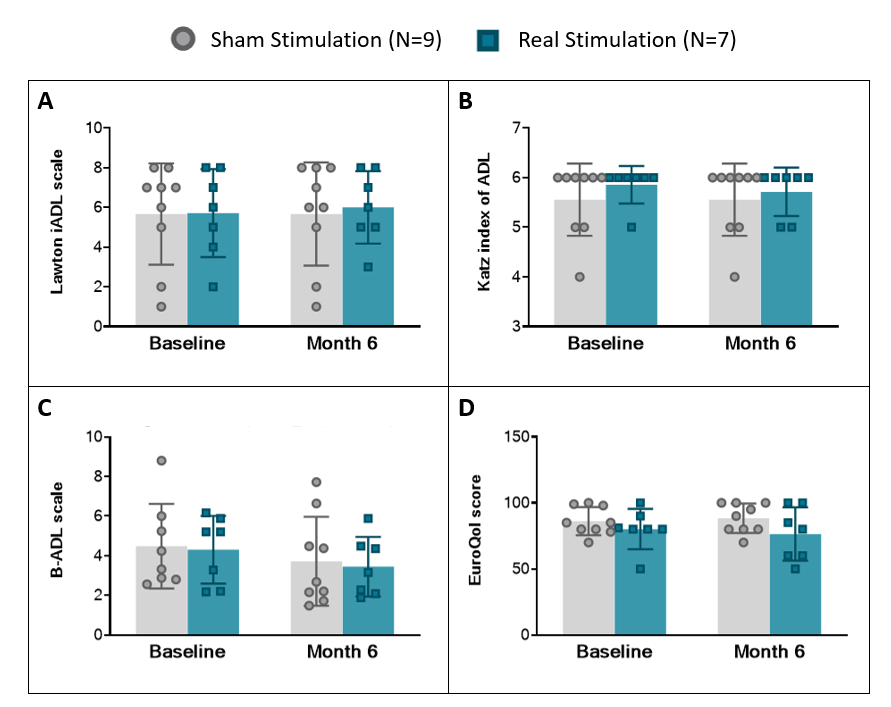


Supplementary Figure 1. Changes in secondary outcome scores over time, including all quality-of-life measurements. (A) Comparison of iADL scores at baseline and after 6 months of treatment. (B) Comparison of ADL scores at baseline and after 6 months of treatment
